# Corneal crosslinking efficacy in patients with keratoconus under 18 years of age

**DOI:** 10.5935/0004-2749.20210046

**Published:** 2021

**Authors:** Evandro Ribeiro Diniz, Júlia Carvalho Barbosa, Raíza Jacometti, Renata Tavares Silva Souza, Fábio Nishimura Kanadani

**Affiliations:** 1 Instituto de Olhos Ciencias Médicas, Belo Horizonte, MG, Brazil; 2 Santa Casa de Misericórdia de Belo Horizonte, Belo Horizonte, MG, Brazil; 3 Mayo Clinic, Jacksonville, Florida, EUA

**Keywords:** Keratoconus/diagnosis, Keratoconus/drug therapy, Cornea, Corneal diseases, Corneal topography, Collagen/metabolism, Ultraviolet rays, Cross-linking reagents/therapeutic use, Riboflavin/therapeutic use, Visual acuity, Adolescent, Ceratocone/diagnóstico, Ceratocone/tratamento farmacológico, Córnea, Doenças da córnea, Topografia da córnea, Colágeno/metabolismo, Raios ultravioleta, Reagentes para ligações cruzadas/uso terapêutico, Riboflavina/uso terapêutico, Acuidade visual, Adolescente

## Abstract

**Purpose:**

Keratoconus presents certain specificities in pediatric patients compared with adults. The greatest challenge is because the disease is typically more severe and progresses faster in children. This retrospective study aimed to report crosslinking procedure in patients under 18 years of age and their follow-up for at least 24 months after the procedure.

**Methods:**

Overall, 12 eyes from 10 patients were studied and data, such as visual acuity with and without correction, maximum keratometry, corneal thickness, foveal thickness, and endothelial microscopy, were assessed at both preoperative and postoperative visits. Corneal crosslinking was performed in all patients.

**Results:**

A tendency toward reduced K_max_ and improved Corrected Distance Visual Acuity at all postoperative moments. Only one of the 12 eyes exhibited increased K_max_ of more than 1 D during a time frame longer than 12 months. Regarding pachymetry, a tendency for corneal thinning was observed in the first four months after surgery.

**Conclusion:**

Encouraging results were obtained regarding the stabilization of the disease, progression, and procedural safety, corroborating to other authors’ findings. The significance of early diagnosis and short-term follow-up were highlighted.

## Abstract (pt)

**Objetivo:**

O ceratocone na população pediátrica apresenta algumas particularidades em relação à população adulta. O maior desafio é devido à doença ser geralmente mais severa e rapidamente progressiva em crianças.

**Métodos:**

Este artigo utiliza uma análise retrospectiva para relatar o uso do crosslinking em jovens menores de 18 anos e sua evolução pelo menos 24 meses após o procedimento. Foram estudados 12 olhos de 10 pacientes, e dados como acuidade visual com e sem correção, ceratometria máxima, espessura corneana, espessura foveal e microscopia endotelial avaliados no pré e pós-operatórios. O crosslinking corneano foi realizado em todos os pacientes pelo mesmo cirurgião.

**Resultados:**

Observou-se uma tendência de redução do valor do K_max_ e melhora da acuidade visual corrigida em todos os momentos de pós operatório. Com relação à paquimetria, observou-se afinamento corneano do ponto mais fino, nos primeiros quatro meses de pós-operatório.

**Conclusão:**

Resultados encorajadores foram obtidos com relação à estabilização da doença, progressão e segurança do procedimento, corroborando com as conclusões de outros autores. A importância do diagnóstico precoce e do acompanhamento a curto prazo do paciente deve ser destacada.

## INTRODUCTION

Keratoconus (KC) is a progressive condition wherein the cornea attains a conic shape after a non-inflammatory thinning of the stroma, resulting in astigmatism, myopia, and variable visual impairments. A reduced number of cross-linking (CXL) among collagen fibers and a higher pepsin expense level have been suggested as the cause of corneal susceptibility to KC. KC typically starts during puberty, and is progressive until the third to fourth decades of life. However, it may begin earlier and keep progressing until more advanced ages^(1)^. In addition, atopy, the act of rubbing eyes, and genetic inheritance (6%-8% of the described cases have a positive family history) are associated with this pathology^(2)^.

KC in the pediatric ages and young patients is significantly different than in adults. It is generally underdiagnosed because the complaints are less frequent as are the routine ophthalmic visits. Nevertheless, it tends to be more progressive and severe in younger patients. However, there are no well-defined treatment protocols for the pediatric population^(3-5)^.

KC management in pediatric patients is typically based on the existing options for adults, such as visual improvement by wearing glasses, contact lenses, intrastromal ring implant, and corneal transplant depending on the disease stage. KC diagnosis before adulthood is a risk factor for poor progression of the disease, with a higher probability of corneal transplant. Among the pediatric population, KC accounts for an overall 15% to 20% of keratoplasties^(3)^.

Collagen CXL involves a photopolymerization reaction that induces biochemical and microstructural changes in the corneal stroma, strengthening the connections among collagen fibers using riboflavin activated by ultraviolet radiation A (UVA)^(6)^. It has been increasingly used as a therapeutic option and is the only proven option capable of changing the disease progression^(4)^.

Several studies have been published that assessed the technique, and a standard protocol for CXL use has been established (the Dresden’s protocol)^(3,4,6)^. Moreover, the standard protocol has been variously modified because of the technique’s success, such as the use of accelerated and transepithelial CXL.

Most studies have assessed CXL treatment for KC in adult patients, and only few studies have addressed the feasibility of this technique in young patients. Upon literature review, a study was identified that conducted a 10-year follow-up of patients with keratoconus under 18 years of age who underwent CXL and demonstrated the ability of CXL to slow down KC progression in pediatric patients and improving functional performance, with KC stability recorded in approximately 80% of patients^(7)^.

In the present study, we investigated whether CXL stabilizes KC progression in patients under 18 years of age.

## METHODS

Our study is a chart review of 12 eyes (N=12) from 10 patients under 18 years of age who underwent corneal collagen CXL because of progressive KC at a private ophthalmic clinic in Belo Horizonte, Minas Gerais, Brazil, from 2013 to 2018.

All patients had a prior diagnosis of KC based on clinical, biomicroscopic signs, and per the Rabinowitz criteria based on placid anterior curvature values^(1,5)^. The diagnosis was confirmed through sequential follow-up with corneal tomography (OCULUS Pentacam^®^, Germany).

Inclusion criteria were progressive KC (defined as the increase of the topographic keratometry in the corneal apex higher or equal to 0.75 D over a period of 6 months or higher than 1.0 D in 1 year), corneal thickness in its thinnest point of at least 400 µ, absence of other anterior segment diseases as well as other systemic pathologies that could interfere with corneal scarring, attendance at all semiannual appointments, and postoperative period of at least 2 years.

Patients who had an intrastromal ring implant and those whose missed follow-ups were excluded from the study.

The following relevant preoperative and postoperative data were studied: visual acuity with and without correction (logMAR), corneal thickness (OCULUS Pentacam^®^), maximum keratometry (Kmax: the most curved part of the cornea, OCULUS Pentacam^®^), foveal thickness (macular optical coherence tomography, Heidelberg SPECTRALIS^®^), analysis of corneal endothelial cells (specular microscopy, Konan Non-Con Robo Specular Microscope). The Amsler-Krumeich classification was used to determine the disease severity. Patients using contact lenses were advised to suspend their use at least 2 weeks before the examination.

The surgical procedure was performed under sterile conditions by the same surgeon (ERD) in accordance with the Dresden protocol^(4)^ as follows: topical anesthesia with proximetacain1 drop 5 min before the procedure and 1 drop immediately before the procedure, removal of 8-10 mm of the corneal epithelium, application of riboflavin (0.1% riboflavin-5-phosphate and 20% dextran T-500) 30 minutes before and every 5 minutes during radiation (30-minute exposure to 370 nm UVA at 3 mW/cm^2^). No general anesthesia or sedation was performed.

During the postoperative period, topical antibiotic (moxifloxacin) and steroids (dexamethasone) four times a day were prescribed for 10 days, and patients were advised to wear therapeutic contact lenses for 5 days.

All patients and their parents or guardians were previously informed of the risks and benefits of the procedure and informed consent forms were obtained from all of them per the tenets of Helsinki.

Numerical variables were presented as mean ± standard deviation. The mean and standard deviation were considered more intuitive because the Friedman test uses ranks rather than the median for comparison of the means before the procedure and after the Friedman test was performed. Although the variables were continuous, they cannot assume the normality of data (mainly owing to the sample size); therefore, ANOVA could not be used. Multiple comparisons were evaluated based on the least significant difference. The analysis was performed using the software R, version 3.4.3, and adopted a level of significance of 5%.

This study was approved by the Research Ethics Committees of Faculdade Ciências Médicas de Minas Gerais and Hospital Universitario Ciências Médicas based on the Resolution CNS 196/96 followed by the technical report 2.740.371 (CAAE:90113418.0.0000.5134).

## RESULTS

Twelve eyes from 10 patients were included. The mean age at the time of the procedure was 14.42 ± 2.27 years and four were girls. All demographic study data are presented in table1.

**Table 1:** Demographic characteristics of patients subjected to CXL

Patient	Eye	Age CXL was performed (in years)	Sex	Amsler-Krumeich classification
1	RE	17	F	2
2	RE	15	F	2
3	RE	16	M	4
4	RE	13	M	4
5	LE	17	F	4
6	LE	10	M	2
7	RE	12	M	3
7	LE	14	M	2
8	LE	17	M	2
9	LE	15	M	3
9	RE	15	M	2
10	RE	12	F	4

RE= Right eye; LE= Left eye; F= female; M= Male.

Tables 2 and 3 present the mean and standard deviation of each analyzed variable from the preoperative period until the semiannual follow-up appointments up to 5 years after surgery.

**Table 2:** Mean and standard deviation for each variable before procedure and during four postoperative assessments

Variable	Pre	6 months post	12 months post	18 months post	24 months post
Kmax^*^	57.15 ± 7.68	55.14 ± 7.35	54.60 ± 7.66	53.66 ± 7.19	54.31 ± 7.19
CDVA	0.31 ± 0.19	0.19 ± 0.14	0.17 ± 0.14	0.18 ± 0.14	0.26 ± 0.19
Pachymetry	457.91 ± 33.43	421.50 ± 36.96	421.27 ± 42.11	428.11 ± 52.57	428.45 ± 51.56
Foveal thickness	231.27 ± 22.04	234.44 ± 18.25	235.00 ± 20.56	220.00 ± 10.17	233.71 ± 37.30
UDVA	0.72 ± 0.44	0.50 ± 0.27	0.46 ± 0.32	0.57 ± 0.35	0.52 ± 0.32
Endothelial cells	2928.41 ± 407.50	2917.67 ± 549.33	3014.10 ± 513.70	2997.20 ± 571.24	3376.57 ± 550.71

**Table 3:** Mean ± standard deviation for each variable during the last five postoperative assessments

**Variable**	**36 months post**	**42 months post**	**48 months post**	**54 months post**	**60 months post**
Kmax	51.50 ± 5.89	52.93 ± 5.75	51.35 ± 2.05	51.97 ± 6.17	51.65 ± 7.28
CDVA	0.33 ± 0.25	0.17 ± 0.12	0.20 ± 0.14	--	0.40^*^
Pachymetry	419.75 ± 8.30	398.00 ± 17.09	404.00 ± 14.14	407.00 ± 9.90	418.00 ± 11.31
Foveal thickness	236.00 ± 17.35	244.00 ± 25.12	--	--	224.00^*^
UDVA	0.30 ± 0.00	0.48 ± 0.34	0.30^*^	1.00^*^	0.50 ± 0.71
Endothelial cells	3000.00 ± 765.57	2974.67 ± 828.63	2779,00^*^	--	2902.00^*^

* = n=1; --= no data.

**Graph 1:**
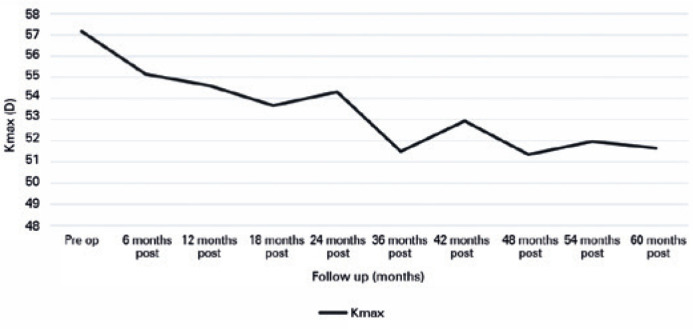
Preoperative and postoperative Kmax evolution of patients undergoing crosslinking.

Comparing the Kmax results, a statistically significant difference was observed at all visits (*p*=0.002), except for Kmax difference between preoperative and 6-month postoperative periods and Kmax difference between 12 and 18 months after the procedure (Graph 1). A tendency toward the reduction in Kmax mean and its standard deviation was noted from the preoperative period to 5 years after surgery.

**Graph 2:**
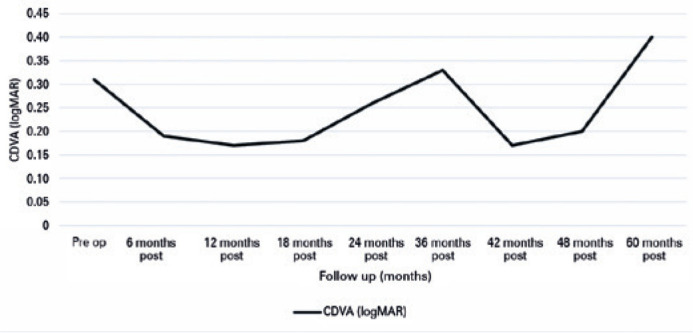
Preoperative and postoperative CDVA evolution of patients undergoing crosslinking.

Corrected distance visual acuity (CDVA) exhibited a significant difference (*p*=0.006) when compared with the preoperative mean at all postoperative assessments (Graph 2). The CDVA mean improved from 0.30 to 0.17 (logMAR) during the first year after surgery. This represented a gain of approximately two lines of vision. A crucial point to highlight was that no vision loss was observed in any of the analyzed patients.

Furthermore, a statistically significant difference (*p*=0.023) was observed on comparison between the preoperative pachymetry at the thinnest point or mean and the first four postoperative assessments. No statistically significant difference was noted regarding the foveal thickness (*p*=0.961) between pretreatment and 24-month follow-up time point. Regarding uncorrected distance visual acuity (UDVA) and endothelial evaluation, the Friedman test could not be performed because only two patients had the complete data.

Ectasia progression halt, and consequently therapeutic efficiency, was considered in those patients whose Kmax did not exhibit an increase higher or equal to 1 D during the 12-month postoperative period. Table 4 displays the observed keratometric differences during the postoperative periods, revealing that only one of the 12 eyes presented a Kmax increase higher than 1 D in the time frame longer than 12 months.

**Table 4:** K_max_ variation during postoperative periods

Patient	18-24 months (6-month difference)	12-24 months (12-month difference)	6-24 months (18-month difference)
1	-0.1	-0.2	-1.3
2	-	-	-
3	-	-1.9	-3.2
4	-1.5	-1.0	-1.0
5	-0.3	-0.3	-0.5
6	-	0.0	-0.2
7	-0.5	-2.8	-4.3
7	0.6	-	1.3
8	-0.1	0.5	-0.2
9	-0.5	-1.4	-2.0
9	0.0	-0.9	-1.9
10	-0.4	0.6	-3.4

## DISCUSSION

The significance of using CXL for treating KC in children has been increasing over the years. However, the literature reveals no consensus regarding the indications or management of the procedure in pediatric patients.

The corneal biomechanical stiffness in youngsters is more fragile, which may contribute to faster progression of the disease in that age group^(3,8)^. The treatment of allergic diseases may be more challenging in these patients owing to a higher occurrence and the difficulty in controlling habits. Furthermore, the use of contact lenses as a therapeutic option is less tolerated by children and a penetrating transplant is more likely to be rejected^(9)^.

Some previous studies have assessed corneal CXL results in the pediatric population. Other studies have concluded that after the procedure there have been an increase in visual acuity with disease stabilization^(2,10,11)^, which is concordant with the findings observed in the cases of this study.

An Irish retrospective study involving patients up to 18 years of age analyzed 25 eyes treated with CXL and followed up for 1 year^(8)^. They revealed a stability of uncorrected visual acuity, refractive indices, and keratometric values. Regarding pachymetry measurement at the thinnest point of the cornea, a significant reduction in the mean values during the first 6 months and recovery to preoperative values after 1 year were observed. In the present study, we observed a statistically significant reduction in total corneal thickness in the first 2 years of follow-up. Another study included 40 eyes of patients up to 18 years of age undergoing CXL^(2)^. CXL was observed to improve UCVA and best spectacle-corrected visual acuity (BSCVA) in the study patients, most probably by significantly reducing the corneal asymmetry and corneal as well as the overall wavefront aberrations. However, the endothelial cell counts did not change significantly. In our study, the endothelial cell count was stable, corroborating the literature findings^(2,8)^.

In the studied cases, the usage of UVA radiation in the energy level used for CXL did not cause structural or functional damage to the macula, based on the foveal thickness measured using the spectral domain optical coherence tomography (OCT), thereby reinforcing the safety of the procedure. It was demonstrated that CXL in the pediatric population could result in corneal flattening, with the reduction of Kmax during the postoperative period until the end of the 5-year follow-up. Seiler *et al.* assessed patients who underwent CXL over a 10-year follow-up period and studied parameters related to the corneal remodeling process^(12)^. Out of 45 eyes, 3 revealed a continuous corneal flattening process, with a decrease in keratometric values not associated with stromal scarring. Such cases differ from the long-term standard outcomes of corneas that have undergone CXL and present a flattening of approximately 2.5 D during the first three postoperative years, followed by a stability phase. The cases studied by this author are also different from the eyes that evolve to a strong flattening owing to stromal opacities. Patients who evolved to a corneal flattening had a beneficial effect of myopia reduction, with a tendency to emmetropia in the first year.^(12)^ However, constant corneal flattening indicates that this process may continue indefinitely and cause progressive hypermetropia^(12)^.

A cohort study analyzed the use of CXL for treating KC in different age groups^(13)^. They reported that cones in children tend to be more accented and a greater improvement in visual acuity was observed among children compared with adolescents and adults who have undergone the same procedure. Therefore, CXL was considered equally safe in all age groups.

Even though epithelial abrasion has been the treatment choice in children, accelerated CXL and transepithelial technique have been studied. A prospective study by an interventionist on 30 eyes from 18 patients who underwent the accelerated method concluded that the highest energy and the shortest treatment time (9 mW/cm^2^ for 10 minutes) could be a better option for children because after 2 years of follow-up improvement in visual acuity, less cylindrical refractive error, and excellent keratometric values were observed^(14)^. Another study demonstrated the efficacy and safety of the accelerated method on 28 eyes of pediatric patients and reinforced the findings described above^(15)^.

Twenty-two eyes that underwent transepithelial CXL technique with the adjuvant use of ethylenediamine tetraacetic acid (EDTA) and trometamol (they increase the epithelial permeability to hydrophilic macromolecules) revealed encouraging results during 1-year follow-up^(16)^. On the other hand, Buzonnetti et al. demonstrated that the interruption of the KC progression was not as efficient as the traditional method despite the visual acuity improvement observed in patients subjected to transepithelial CXL^(17^).

Nevertheless, the ideal moment for performing the procedure is another matter of debate. Most authors point out that the best time would be when the first signs of progression are identified. However, others advocate the benefit of using CXL as soon as the KC diagnosis is made in the pediatric population^(6,16,18)^.

According to Chatziset al., the effects of CXL in children and adolescents may not last as long as in adults, thereby necessitating further postoperative assessments^(18)^. Their study evaluated 46 eyes and most exhibited significant keratometry (Kmax) reduction during the first 24 months, but losing its significance after 36 months^(18)^.

A longitudinal cohort study assessed 62 eyes from 47 patients during a 10-year follow-up and analyzed the efficacy and safety of CXL in the pediatric population, similar to our study^(18)^. They observed a statistically significant reduction in Kmax in the sixth month of treatment up until the eighth year of follow-up, with improvement in visual acuity results for a long period^(18)^. At 36 months, four eyes exhibited KC progression and a re-treatment was performed with Kmax stabilization. Two eyes from two patients were treated using anterior lamellar keratoplasty owing to low vision corrected by contact lenses, even with a stable cone after CXL^(18)^. The study revealed a 24% KC progression throughout the follow-up^(18)^. They reported that the CXL’s capacity to reduce KC progression was approximately 80% throughout 10 years in the pediatric population^(18)^. Accordingly, the results at 7 to 10 years after treatment revealed that collagen turnover induces the CXL’s loss of effect with KC instability and progression, and the procedure would have to be repeated in 25% of cases^(18)^. In our study, only one patient had a Kmax increase higher than 1 D during the time frame longer than 12 months, with no deterioration in visual acuity or need for re-treatment.

Nonetheless, this study is not free of limitations. Despite the small number of studied eyes, all of them had a minimum follow-up of 2 years, with some reaching up to 5 years. Another limitation is the retrospective design, where a lack of documented information could lead to loss of information. The Friedman test, for example, was not performed for the UDVA and the endothelial cell count because of the lack of data at all visits.

Treatment with CXL was effective in 11 of the 12 eyes studied, with disease stabilization in 91.6% of cases. Only one patient had a Kmax increase higher than 1 D during the follow-up, but without decreased visual acuity and keratometric stability in the subsequent years. In addition, CXL can be considered a safe procedure in the pediatric population because it did not cause any vision loss at any time and no complications were encountered during or after the procedure.

Generally, CXL in the pediatric population could be considered a safe and viable procedure because the treatment was performed under topical anesthesia without the need for previous sedation. This form of anesthesia would facilitate the presence of a parent or guardian in the operating room for children with severe anxiety.

In conclusion, clinical experiences suggest that KC in children typically evolves rapidly with significant visual impairment. Therefore, the control that corneal CXL provides over ectasia progression becomes paramount in this age group, helping to avoid penetrating keratoplasty. Nevertheless, the significance of early diagnosis and short-term follow-up (every 3 months, according to most authors) need to be highlighted^(2,3)^.

Nevertheless, further studies are needed to assess whether CXL can be as effective in the pediatric population as in adults in the long run, as well as analyze the possibility of re-treatment. In addition, the use of higher energy than described in the standard protocol to increase the efficacy of treatment needs to be discussed^(3)^.
